# Facial subcutaneous emphysema of late onset after frontal sinus fracture

**DOI:** 10.1590/S1679-45082016AI3532

**Published:** 2016

**Authors:** Andreia Filipa Miranda Mota, Virgínia Machado, Sofia Peças, Alexandra Emílio, Eduarda Marisa Vicente

**Affiliations:** 1 Centro Hospitalar de Setúbal, EPE, Setúbal, Portugal.

**Figure 1:**
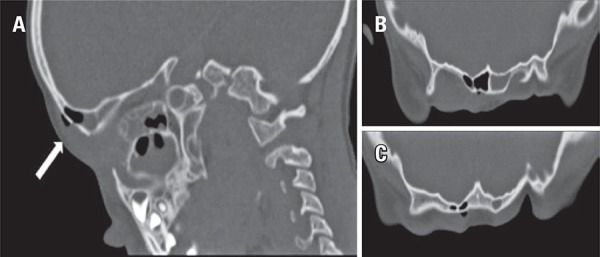
(A) Right parasagittal plane, (B) supraorbital transverse plane and (C) frontal transverse plane

A 5-year-old boy with previous history of frontal sinus fracture, 2 years ago, with conservative treatment and no current surgical follow-up. Upon admission, he had sudden onset of fever, severe swelling over the right eyelid and supraciliary region, and painful ocular movement. Physical examination showed no other alterations, therefore, excluding neurological compromise. Laboratory tests revealed raised inflammatory markers. For staging the extent of inflammation and exclude intracranial complications a computed tomography scan of the head and orbits was performed and revealed right frontal and periorbital swelling extending to postseptal area without optic nerve involvement. In addition, we observed subcutaneous emphysema around a break in the continuity of the anterior wall of frontal sinus table and a process of acute pansinusitis with air-fluid levels involving maxillary, ethmoid, sphenoid and frontal sinuses; there was no abscess formation. We admitted the diagnosis of orbital cellulitis and facial subcutaneous emphysema due to pansinusitis. Facial subcutaneous emphysema has been reported as an acute complication of head trauma.^(^ ^1^ ^-^ ^4^ ^)^However, in this case, it was caused by a late complication of past bone fracture, probably triggered by an acute process of frontal sinusitis, which is a rare complication.

Subcutaneous emphysema is just one of the complications associated with pediatric head trauma, which also includes seizures, epidural, subdural or intracranial haemorrhage, cerebral veins and sinus thrombosis, cranial nerve injury, cerebrospinal fluid fistulas and pneumocephalus.^(^ ^1^ ^,^ ^2^ ^,^ ^5^ ^-^ ^7^ ^)^ The majority of these complications occur mainly in acute phase. Although, pneumocephalus and seizures can also be a late complication. Physicians should be aware of both acute and long-term complications of head trauma in order to ensure prompt and adequate intervention, and therefore, guarantee better outcomes.^(^ ^1^ ^-^ ^8^ ^)^
